# Quantitative Sensory Testing in an Observational Cohort of Adults With Chronic Low Back Pain

**DOI:** 10.1002/jsp2.70103

**Published:** 2025-08-19

**Authors:** Michael J. Schneider, Carol M. Greco, Amanda M. Acevedo, Kevin M. Bell, Jessa Darwin, Anthony Delitto, Nathan E. Dodds, John M. Jakicic, Gina P. McKernan, Charity G. Patterson, Paul A. Pilkonis, Sara R. Piva, Gwendolyn A. Sowa, Nam V. Vo, Lan Yu, Ajay D. Wasan

**Affiliations:** ^1^ Doctor of Chiropractic Program, University of Pittsburgh School of Health and Rehabilitation Sciences Pittsburgh Pennsylvania USA; ^2^ Clinical and Translational Science Institute, University of Pittsburgh Pittsburgh Pennsylvania USA; ^3^ Department of Psychiatry University of Pittsburgh School of Medicine Pittsburgh Pennsylvania USA; ^4^ Department of Physical Therapy University of Pittsburgh School of Health and Rehabilitation Sciences Pittsburgh Pennsylvania USA; ^5^ Behavioral Research Program, National Cancer Institute Rockville Maryland USA; ^6^ Department of Bioengineering University of Pittsburgh Swanson School of Engineering Pittsburgh Pennsylvania USA; ^7^ Department of Physical Medicine and Rehabilitation University of Pittsburgh School of Medicine Pittsburgh Pennsylvania USA; ^8^ Office of the Provost, University of Pittsburgh Pittsburgh Pennsylvania USA; ^9^ Department of Internal Medicine University of Kansas Medical Center Kansas City Kansas USA; ^10^ Ferguson Laboratory for Orthopaedic and Spine Research, Bethel Musculoskeletal Research Center, Department of Orthopaedic Surgery University of Pittsburgh School of Medicine Pittsburgh Pennsylvania USA; ^11^ Department of Orthopaedic Surgery University of Pittsburgh School of Medicine Pittsburgh Pennsylvania USA; ^12^ Department of Pathology University of Pittsburgh School of Medicine Pittsburgh Pennsylvania USA; ^13^ McGowan Institute, University of Pittsburgh Pittsburgh Pennsylvania USA; ^14^ Department of Medicine University of Pittsburgh School of Medicine Pittsburgh Pennsylvania USA; ^15^ Department of Anesthesiology and Perioperative Medicine University of Pittsburgh School of Medicine Pittsburgh Pennsylvania USA

**Keywords:** chronic low back pain, conditioned pain modulation, pressure pain threshold, quantitative sensory testing, temporal summation

## Abstract

**Background:**

Quantitative Sensory Testing (QST), also known as psychophysical testing, includes standardized methods for assessing humans' perceptions of different types of sensory stimuli and their associated pain thresholds. QST results can be used to estimate altered or atypical sensory processing and thus can be useful for determining pain mechanisms such as nociplastic or central nervous system‐mediated pain. The University of Pittsburgh Mechanistic Research Center, entitled, “Low Back Pain: Biological, Biomechanical, Behavioral Phenotypes (LB^3^P),” is part of the National Institutes of Health's Helping to End Addiction Long‐term Initiative. LB^3^P conducted a prospective, observational cohort study to identify phenotypes of over 1000 participants with cLBP. QST was conducted on these participants as part of comprehensive data collection. This article reports on the results of the QST procedures performed at the initial in‐person enrollment visit.

**Methods:**

Four QST procedures were administered to participants of the LB^3^P study at their enrollment visit: (1) Pressure Pain Thresholds (PPT) over the participant‐reported site of lumbar pain (paraspinals) and a control site (trapezius) using an analog algometer; (2) Temporal Summation (TS) over the lumbar pain and control sites (forearm) using a Neuropen with a 40‐g monofilament; (3) Conditioned Pain Modulation (CPM) using a cold water (5°C) immersion tank; and (4) Cold Water Tolerance time. A subset of LB^3^P participants was excluded from the CPM and cold‐water immersion procedures due to medical comorbidities such as cardiovascular disease and diabetic neuropathy. Means and standard deviations (SDs) were calculated from three trials of PPT and TS, two trials of CPM, and one trial of cold‐water immersion time. TS was calculated by subtracting the numeric pain scores (0–10 scale) of the first from the 10th pinpricks. CPM was calculated by subtracting the mean trapezius algometer readings during the PPT procedure from those of the trapezius PPT during cold‐water immersion.

**Results:**

The final cohort of QST participants was 999 adults. The mean/SD of lumbar and trapezius PPTs was 4.6 (2.4) and 4.4 (1.9) kg/cm^2^, respectively. The mean/SD of lumbar and forearm TS was 1.6 (2.0) and 1.2 (1.8). Lingering pain after the 10th pinprick (after‐sensations) was reported by 19.3% and 15.6% of participants after a series of 10 pinpricks was applied to the lumbar pain site and control site, respectively. The mean/SD CPM was 0.9 (1.2) with a wide range of CPM values from −2.9 to 5.9. The cold‐water tolerance test resulted in a bimodal distribution, with 83% of participants having an average immersion time of 30 s and the remaining 17% reaching the maximum immersion time of 180 s.

**Conclusions:**

QST data were collected from a large cohort of individuals with cLBP who participated in the LB^3^P observational study. The QST results provide reference values for persons living with cLBP.

## Graphical abstract

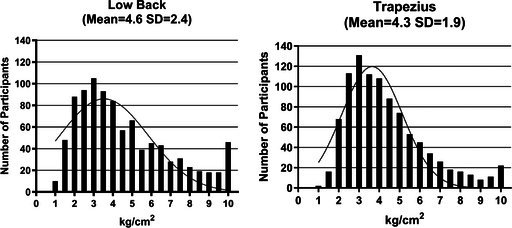

## 1 Introduction

The International Association for the Study of Pain (IASP) defines pain as “An unpleasant sensory and emotional experience associated with, or resembling that associated with, actual or potential tissue damage.” [1] The IASP differentiates between pain and nociception, stating that pain cannot be attributed solely to the activation of sensory neurons. Pain is further classified by the IASP into three categories: nociceptive, neuropathic, and nociplastic.

Nociceptive pain is the normal physiological response to activation of sensory receptors from tissue damage or inflammation and can arise from nociceptors in the visceral or musculoskeletal tissues. An example of nociceptive low back pain (LBP) is an acute tear of the annulus fibrosus of a lumbar intervertebral disc. The outer third of the annular fibers is richly innervated with nociceptors, which cause localized LBP when activated.

Neuropathic pain results from abnormal neural activity caused by a lesion or disease of the somatosensory nervous system, either in the peripheral or central nervous system (CNS). An example of peripheral neuropathic LBP is radiculopathy caused by extruding intervertebral disc material that mechanically and chemically irritates the exiting lumbar nerve roots. In this case, the person is likely suffering from two types of pain: (1) nociceptive pain from activation of the sensory receptors in the annular fibers of the intervertebral disc; and (2) neuropathic pain from the disc lesion that mechanically and chemically activates the nerve.

Nociplastic pain (also known as central sensitization) is defined as pain that arises from altered neural processing of sensory stimuli with or without evidence of actual tissue damage that is causing activation of peripheral nociceptors or a lesion within the somatosensory system. The mechanism of nociplastic pain is thought to be an amplification of neural processing either peripherally or centrally that elicits hypersensitivity. A classic example of nociplastic pain is the widespread, multisite pain and associated symptoms seen in individuals with fibromyalgia syndrome [2, 3, 4].

There is clinical value in knowing whether central sensitization is contributing to a patient's cLBP condition, with nociplastic pain as the dominant pain mechanism [5]. However, it is difficult to measure the underlying neurophysiological processes of nociplastic pain in the typical clinical setting. Quantitative sensory testing (QST) procedures have been used in the research setting to identify altered neurophysiological processing of nociceptive information (central sensitization) that would suggest the presence of nociplastic chronic (c) LBP.

The goal of QST is to provide quantitative, perception‐based measures of multimodal sensory thresholds using various techniques and procedures. QST can include measures of pressure pain threshold (PPT), temporal summation (TS), conditioned pain modulation (CPM), and noxious cold pain tolerance. QST seeks to characterize nociceptive, neuropathic, and nociceptive aspects of pain; but most often, QST is used to identify and differentiate atypical sensory processing found in nociplastic chronic pain conditions from nociceptive and neuropathic pain [1].

A recent systematic review and meta‐analysis examined the results of 24 studies using common QST measures involving participants with cLBP compared to healthy controls [6]. Alteration in sensory processing was more prevalent in people with cLBP, with significantly lower PPTs at remote, nonlow back sites and increased TS over the lumbar region of participants with cLBP compared to healthy controls. Other studies exploring various QST measures among persons with cLBP have produced mixed results [7, 8, 9, 10].

The purpose of this manuscript is to present descriptive results of a series of QST measures collected as part of a large cLBP cohort study. The University of Pittsburgh's Low Back Pain: Biological, Biomechanical, Behavioral Phenotypes (LB^3^P) Mechanistic Research Center (MRC) is a member of the National Institutes of Health's (NIH) Back Pain Consortium (BACPAC) Research Program—which is part of the Helping to End Addiction Long‐term (HEAL) Initiative. The overall objective of LB^3^P is to perform in‐depth phenotyping of patients with cLBP using a multimodal assessment approach that can inform improved treatments. The LB^3^P performed an observational cohort study of over 1000 people with cLBP [11].

## 2 Methods

### 2.1 Overview

The QST consisted of four procedures, as described below [12, 13, 14, 15, 16, 17, 18, 19]. Prior to initiating these procedures, the examiner first identified a primary pain site and two control sites. The primary pain site was located in the lumbar region based on the participant's response to manual over‐pressure palpation performed in the prone position. The control sites were located over the contralateral trapezius muscle (diagonal from pain site) and the nondominant palmar (or palm side) forearm.

### 2.2 Participants

Participants were 1007 persons with cLBP enrolled in the LB^3^P prospective longitudinal cohort study at the University of Pittsburgh [11]. Eligible participants were English‐speaking adults with cLBP, defined as “back pain (in the space between the lower posterior margin of the rib cage and the horizontal gluteal fold) that persisted at least three months and resulted in pain on at least half the days in the past six months.” [20] Participants were excluded if there they were (1) not identified in the University of Pittsburgh Medical Center (UPMC) Electronic Health Record (EHR) System, (2) participating in a masked intervention study for LBP, and/or (3) had a medical condition that would place the participant at increased risk or preclude them from complying with study procedures [21, 22].

Participants were enrolled by referral from clinicians, research registries, and community announcements between June 2020 and March 2024. The in‐person enrollment visit took place at the University of Pittsburgh Department of Physical Therapy—Clinical and Translational Research Center. Participants were followed remotely for 12 months and were compensated for their participation incrementally at all timepoints.

The QST activities took place near the conclusion of the in‐person enrollment visit that included questionnaires, physical tasks, and collection of bio samples. Participants were told that the four QST procedures would cause some mild discomfort/pain, but that none of the procedures were harmful in any way. Some participants, such as those reporting cardiovascular disease, Raynaud's syndrome, or diabetic neuropathy, were excluded from tests that involved immersion of the hand in cold water. Participants were allowed to opt out of any or all procedures. The number of participants that completed the QST tasks was 645 for CPM, 958 for PPT, 971 for TS, and 653 for the cold‐water tolerance task.

### 2.3 Procedures

The four QST activities were performed in the order described below. Licensed, trained physical therapists conducted the QST tasks and recorded the results on a study tablet (Samsung S5e).

#### 2.3.1 PPT

An analog algometer (Wagner Pain Test FPK25 device) with a 1 cm^2^ rubber tip was applied to the trapezius control site. Pressure was manually increased at a rate of 0.5 kg of force per second (10 kg max, metronome guided) until the participant first reported that the sensation of “pressure” transitioned to “painful.” The PPT is the pressure intensity (in kg/cm^2^) read from the algometer at the transition time point. Three separate measurements were conducted with 60‐s rest intervals between each pressure application. The three trapezius PPT measurements were averaged.

Next, the participant was asked to lie in the prone position for PPT testing of the primary pain site. The tester placed the algometer over the paraspinal lumbar area, and an additional three PPT measurements were conducted over the primary pain site with 60‐s rest intervals between each pressure application. The three lumbar PPT measurements were also averaged.

#### 2.3.2 TS

A Neuropen device with a 40‐g Neurotip was used to apply three sets of 10 calibrated pinpricks to both the control and primary pain sites. The TS test used the participant's dominant palmar forearm as the control site. The primary pain site was the same location as the most painful site identified by the participant in the PPT test. During the TS testing, participants were asked to rate the magnitude of sensations at the primary pain site and the control site, as specified below.

The TS procedure began with the control site. The Neuropen was used to apply 10 identical pinprick stimuli over the dominant palmar forearm at a constant rate of one pinprick per second. After the 10th pinprick, the participant was asked to rate the pain intensity of the first and last pinprick, using a 0–10 numeric pain rating scale. Participants also reported if they experienced any ongoing pain after sensations or “lingering pain” at 15 and 30 s following the 10 stimuli. The tester waited 1 min; then applied the next set of 10 pinpricks over the palmar forearm, using the same procedure. A total of three sets of 10 pinpricks were applied; after each set, the participant rated the pain associated with the first and 10th pinpricks. The presence of lingering pain was assessed after each set of stimuli.

After completing three sets of 10 pinprick stimuli over the palmar forearm, the TS procedure next involved performing three sets of 10 pinprick stimuli over the primary pain site with the participant lying in the prone position. Lingering pain (“after‐sensations”) was also assessed after the sets of pinpricks to the low back area.

The TS values were calculated by subtracting the numeric pain score reported after the first pinprick from the pain score after the 10th pinprick for each of the sets of pinpricks. The three TS values at each site were then averaged.

#### 2.3.3 CPM

The CPM test combined the PPT test using an algometer over the same trapezius (right or left) used during the original PPT procedure as the primary stimulus, with the opposite hand used for the cold‐water immersion as the “distractor” stimulus. The participant's hand was submerged in a 5°C cold water circulating tank (IsoTemp 4100 R20, Fisher Scientific) up to the wrist while the pressure algometer was simultaneously applied over the trapezius. The same PPT procedure described above was followed. The trapezius algometer pressure intensity (in kg/cm^2^) at the transition timepoint to painful sensation was recorded. Following a 2 min rest period, the procedure was repeated. The CPM value was calculated by subtracting the mean of the three PPT measurements from the mean of the two cold‐water immersion PPT measurements.

#### 2.3.4 Cold Water Tolerance

This test measured the length of time participants could tolerate cold pain by keeping their hand immersed up to the wrist in a tank of 5°C circulating cold water. This test was performed at least 5 min after completion of the second cold water immersion of the CPM test. The same side hand was used for this test as was used in the CPM procedure. Participants were asked to keep their hand immersed in the cold‐water tank for as long as they could tolerate, or a maximum of 3 min (180 s), whichever occurred first. The examiner used a timer to record the immersion time in seconds as a measure of cold pain tolerance.

### 2.4 Data Analysis

Measures of central tendency were computed: means and standard deviations (SDs) for continuous data, and percentages or counts for dichotomous data such as lingering pain/aftersensations. The data for the overall group are also presented as histograms to illustrate variability in QST results. Means (SDs) were calculated for each QST procedure and reported for the entire cohort, as well as by sex (male; female) and by age (< 60; ≥ 60 years).

## 3 Results

A total of 1007 individuals (60% female) were enrolled with an average age of 59 ± 17 years. Most participants were non‐Hispanic (90%), White (75%), and 53% attained college or higher education. 54% were married or had a partner, 43% were employed, 38% retired, 41% had annual household income < $50 000, 20% had been off work for more than 30 days due to LBP, 16% had applied for or received disability, and 6% were on worker's compensation. The majority were obese (average BMI of 31.5 Kg/m^2^), 61% had back pain for > 5 years, and pain had been ongoing every or nearly every day in 76% of the sample [21].

The results are presented for the overall group and stratified by sex at birth (male and female) and by age (< 60 and ≥ 60 years) in Table 1. For various reasons described earlier, not all 1007 participants received all four QST procedures.

**TABLE 1:** Quantitative sensory testing results.

Procedure	Total (*N* = 646–971)	Female (*N* = 384–597)	Male (*N* = 260–401)	Age < 60 (*N* = 300–424)	Age ≥ 60 (*N* = 345–575)
Pressure pain threshold (PPT) [Average across three trials; kg/cm^2^]	Mean (SD)	Mean (SD)	Mean (SD)	Mean (SD)	Mean (SD)
Lumbar region	4.6 (2.4)	3.9 (2.0)	5.6 (2.6)	4.2 (2.2)	4.9 (2.5)
Upper trapezius	4.4 (1.9)	3.9 (1.7)	5.1 (2.1)	4.2 (1.9)	4.4 (1.9)
Conditioned pain modulation (CPM) [Cold water PPT—Nonwater PPT; kg/cm^2^]	Mean (SD)	Mean (SD)	Mean (SD)	Mean (SD)	Mean (SD)
Difference in pain pressure threshold between cold water and nonwater trials	0.9 (1.2)	0.9 (1.1)	1.0 (1.3)	0.8 (1.1)	1.1 (1.2)
Cold water tolerance	Mean (SD)	Mean (SD)	Mean (SD)	Mean (SD)	Mean (SD)
Total immersion time (Range 0–180 s.)	56.2 (61.3)	49.1 (57.3)	65.9 (65.0)	56.1 (61.7)	55.9 (60.8)
First pinprick numeric pain rating	Mean (SD)	Mean (SD)	Mean (SD)	Mean (SD)	Mean (SD)
Lumbar region	1.4 (1.7)	1.4 (1.7)	1.5 (1.6)	1.6 (1.7)	1.3 (1.6)
Palmar forearm	1.1 (1.6)	1.1 (1.6)	1.2 (1.6)	1.2 (1.6)	1.1 (1.6)
Temporal summation (Average across three trials; 10th–1st pinprick; numeric pain rating: 0–10)	Mean (SD)	Mean (SD)	Mean (SD)	Mean (SD)	Mean (SD)
Lumbar region	1.6 (2.0)	1.7 (2.0)	1.3 (1.9)	1.3 (1.8)	1.7 (2.1)
Palmar forearm	1.2 (1.8)	1.3 (1.9)	1.1 (1.7)	1.1 (1.7)	1.3 (1.9)
Temporal summation (lingering pain reported postprocedure for any of three trials)	Percent of sample	Percent of sample	Percent of sample	Percent of sample	Percent of sample
Lumbar region	19.3%	21.4%	16.0%	23.9%	15.8%
Palmar forearm	15.6%	17.3%	13.0%	16.5%	14.8%

*Note:* Total number of participants completing QST components varies due to contraindications to cold water immersion, and/or participants declining test procedures. One participant reported sex at birth as intersex and was therefore not included in the male/female comparison.

### 3.1 PPT

**FIGURE 1:**
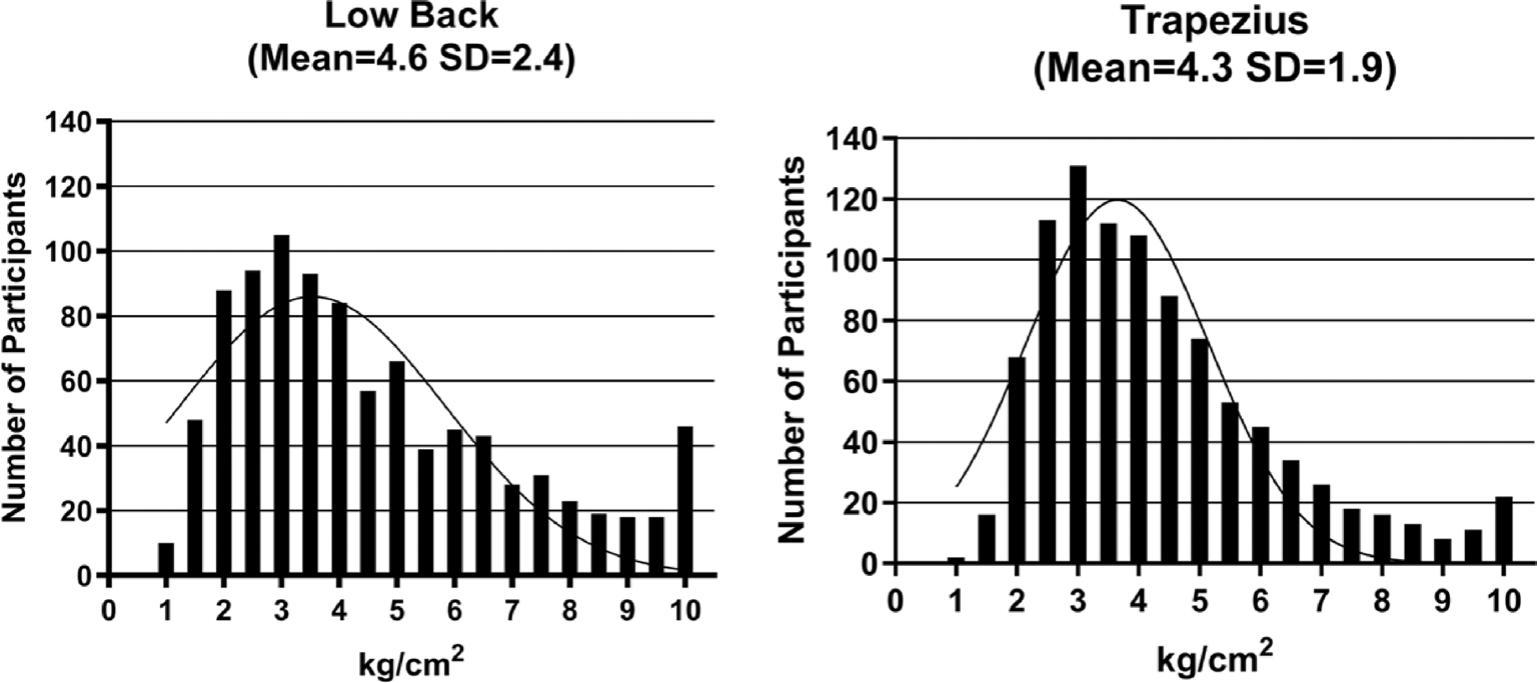
Pressure pain thresholds: X‐axis displays the mean of three applications of pressure using an algometer to elicit the threshold at which patients reported “pain”—not “pressure”—over the low back (site of pain) and trapezius (control site).

We obtained PPT measurements on a total of 958 participants, with the mean/SD of lumbar and trapezius PPTs reported as 4.6 (2.4) and 4.4 kg/cm^2^ (1.9), respectively, in the overall group. Qualitatively, PPT of female participants was lower on average than that of males, and PPT was similar for older and younger participants. Figure 1 depicts the distribution of lumbar and trapezius PPT values in the overall group.

### 3.2 TS

**FIGURE 2:**
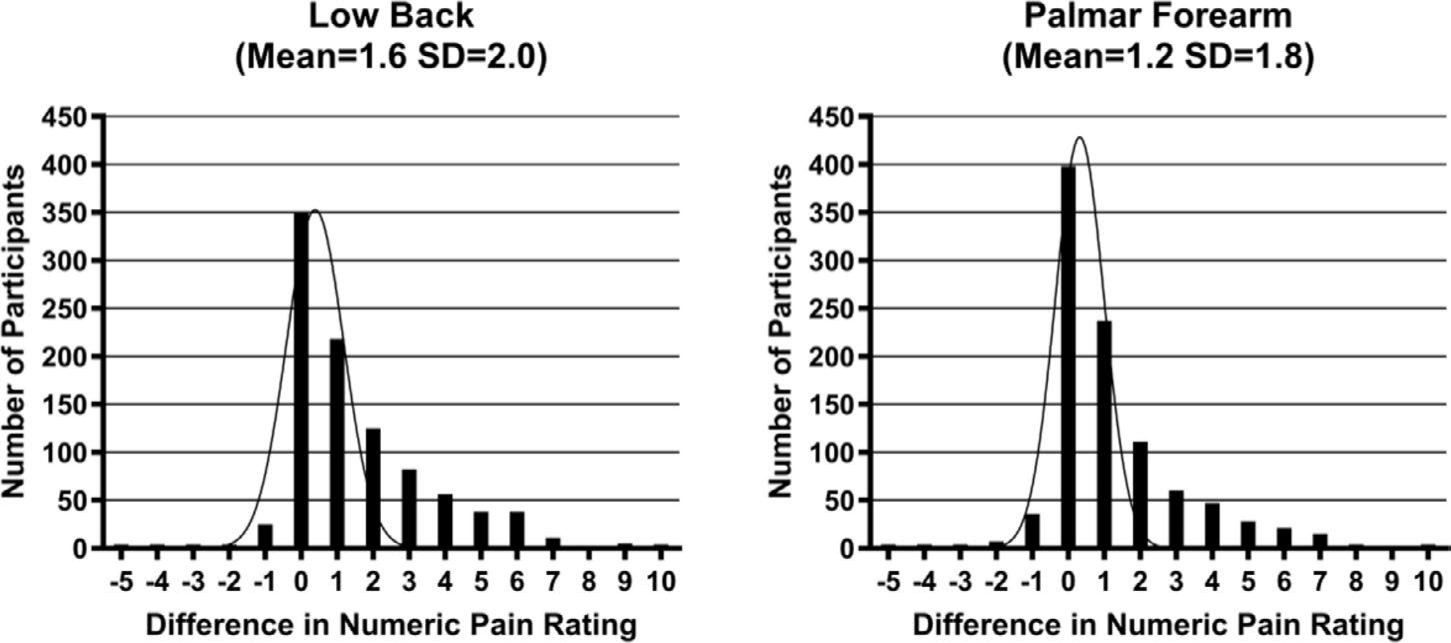
Temporal summation: X‐axis displays the mean differences in pain ratings associated with pinprick stimuli. In each of three trials, participants received a set of 10 pinpricks; participants then numerically rate the pain elicited after the initial (first) and final (10th) pinprick. The 0–10 pain rating of the first pinprick is subtracted from the 0–10 rating of the 10th pinprick, for each trial. The mean values were derived from the three applications of pinpricks, each performed over the low back (site of pain) and the palmar forearm (control site). A negative value indicates that the first pinprick is rated as more painful than the 10th pinprick. A positive value indicates that the 10th pinprick is rated as more painful than the first pinprick.

We obtained TS measurements on a total of 971 participants, with the mean (SD) of lumbar paraspinal (pain site) and palmar forearm (control site) regions reported as 1.6 (2.0) and 1.2 (1.8), respectively. The TS value is calculated as the difference in numeric pain ratings (0–10 scale) when subtracting the pain rating after the first pinprick from the pain rating after the 10th pinprick. Participants' TS values for low back and palmar forearm are shown in Figure 2. Lingering pain (after‐sensations) 15 or more seconds after any of the three lumbar site or three control site trials was reported by 19.3% and 15.6% of participants at the lumbar region and the palmar forearm, respectively. Qualitatively, more females than males and more younger participants than older participants reported lingering pain in the lumbar region (Table 1).

### 3.3 CPM

**FIGURE 3:**
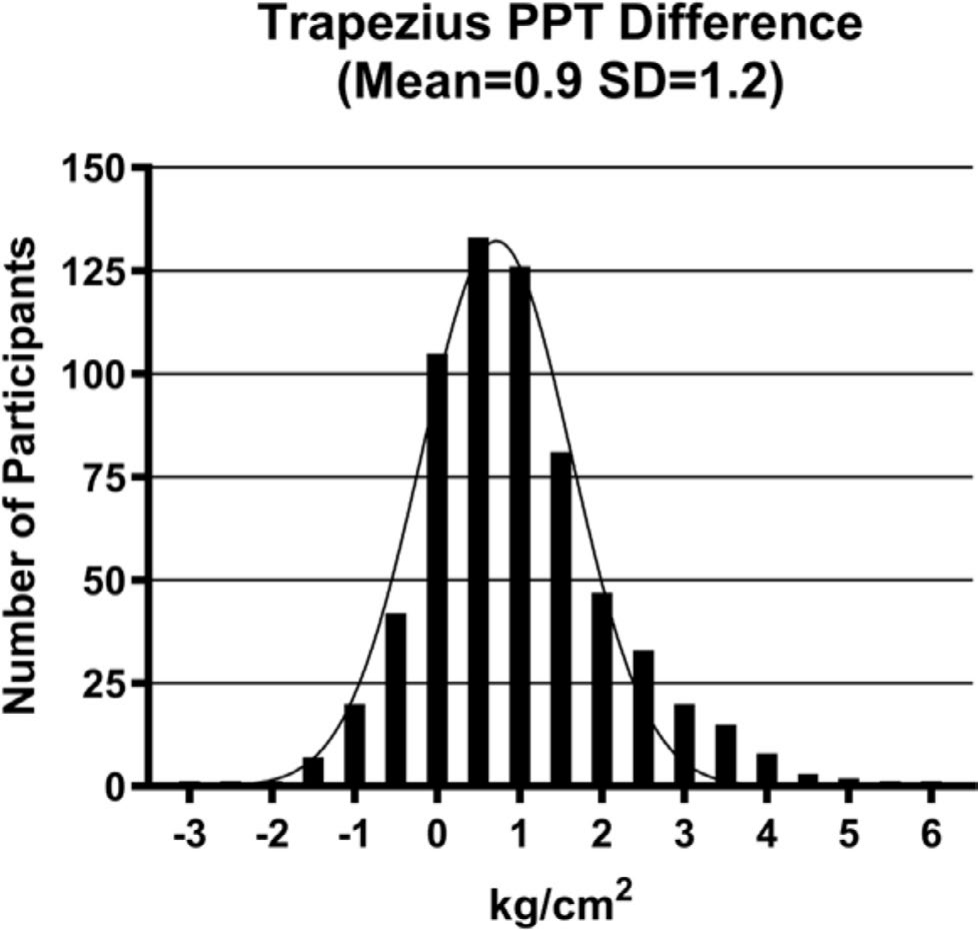
Conditioned pain modulation: X‐axis displays the mean differences in numeric pain elicited during the trapezius pressure pain threshold test (PPT), subtracted from the numeric pain reported during repeated trapezius PPT testing during cold water immersion. Positive values indicate higher pain thresholds during cold water immersion; negative values indicate lower pain thresholds during immersion.

For the 645 persons who engaged in the cold‐water tasks and the PPT tasks, CPM was calculated by subtracting the mean trapezius PPT from the mean cold water immersion trapezius PPT for each participant. For the group overall, the mean (SD) of CPM was 0.9 kg/cm^2^ (1.2). The wide range of CPM values from −2.9 to 5.9 kg/cm^2^ is depicted in Figure 3.

### 3.4 Cold Water Tolerance

**FIGURE 4:**
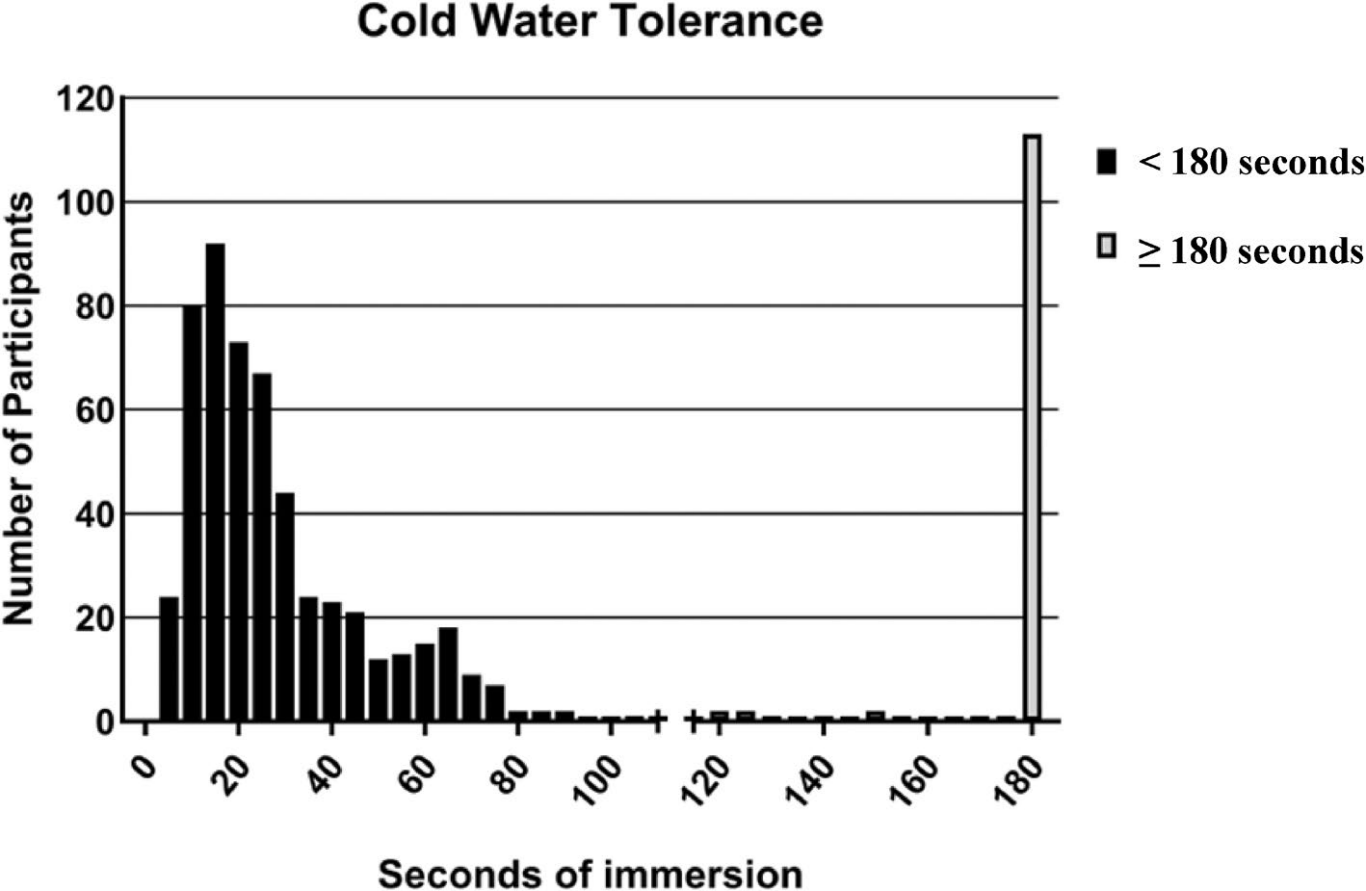
Cold water tolerance: X‐axis displays the number of seconds that participants could tolerate immersion of their hand in cold water. A subset of participants tolerated the maximum allowable immersion time of 3 min, and the majority tolerated less than 2 min.

The mean cold water immersion time for the group overall was 56.2 (61.3) s, with females averaging approximately 18 s less than males. The results of the cold‐water tolerance test had a distinctive bimodal distribution; however, in which 82.8% of participants had an average immersion time of 30.2 s and the remaining 17.2% reached the maximum immersion time of 180 s (Figure 4).

## 4 Discussion

Data were collected within a large prospective cLBP cohort study, LB^3^P, that included QST procedures at participants' primary LBP paraspinal site and other peripheral control locations. This cohort represents a large cross‐sectional sample of participants with cLBP whose characteristics add to the body of cLBP literature. In this paper, we presented the cohort's results for each QST modality, which suggest multiple next steps for future research.

One interesting observation is the mean PPT of about 4.0 kg/cm^2^, which was the threshold for the presence of tender points in the original (1990) classification criteria for a diagnosis of fibromyalgia syndrome [22]. This result suggests that cross‐referencing the PROs data on widespread pain with QST findings may yield a more detailed characterization of the degree of nociplastic pain in the current sample.

PPT and TS values were similar over the lumbar pain and control sites, suggesting concordance between participants' perceptions of their LBP and their overall pain sensitivity. This finding encourages further investigation of the extent to which local characterization of a cLBP syndrome via QST measures is associated with pain processing in the nervous system in general.

Another interesting finding deserving further investigation is that 16%–19% of participants had “lingering pain” after TS testing over the lumbar pain and forearm control sites. Lingering pain has been under‐investigated in the QST literature; and it is only with large samples—such as the present one—that a meaningful percentage of these participants can be identified for further research. It is possible that the intensity of lingering pain is related to nociplastic and/or neuropathic pain processes. Similarly, during CPM, approximately 15% of participants had worsening pain with PPT testing at the control site (trapezius) while their hand was immersed in cold water as a distraction stimulus. CPM refers to the phenomenon of “pain inhibits pain,” (descending antinociceptive system) where the presence of a second noxious stimulus decreases the pain perception from an initial nociceptive stimulus [23]. This finding suggests that certain cLBP participants experience pain facilitation, rather than descending pain inhibition, with CPM.

Finally, there is a clear bimodal distribution of immersion times for cold water testing, with 17% tolerating the full 3 min of allowable immersion. Using the whole sample in a single analytic framework (which is a typical approach in the field) may not be appropriate if we want to understand how the variability in this response is related to clinical pain syndromes. Instead, it may be more prudent to study cold water immersion as a categorical—rather than a continuous—variable in analyses that use QST procedures.

There are limitations in the current study. One limitation was the use of “bedside” tools for QST and not a more comprehensive assessment. The decision to use an abbreviated set of QST procedures was made to enhance feasibility and to reduce participant burden. In addition, the current study's bedside tools have been validated by our group [18], so there is reason for confidence in our procedures. The choice to exclude persons with cardiovascular disease, peripheral neuropathy, and other medical comorbidities such as Raynaud's phenomena from CPM and cold tolerance testing could be considered a limitation depriving us of the opportunity to evaluate pain processing within these groups. However, we decided to err on the side of caution and minimize potential discomfort and harm for these individuals. Another potential limitation is the choices we made concerning the temperature of 5°C for the cold‐water bath and the 180‐s maximum time of cold‐water immersion. For this test, small differences in temperature can have an impact on results [24]. We found that approximately 17% of participants in the cold tolerance test achieved the 180‐s threshold. Using a lower water temperature and a longer time period may have resulted in different and valuable information.

In summary, we present here a general characterization of a large cohort of participants with chronic LBP undergoing bedside QST. Several results suggest questions for more detailed investigation; the size of our sample is an advantage for uncovering novel findings and pursuing further analyses.

## Conflicts of Interest

The authors declare no conflicts of interest.

## Supporting information


**Data S1:** Supporting Information.
