# Supplementary material for: Quantitative Sensory Testing in an Observational Cohort of Adults With Chronic Low Back Pain
Source: JOR Spine. 2025 Aug 19;8(3):e70103. doi: 10.1002/jsp2.70103 (PMC12363403; doi:10.1002/jsp2.70103)
Supplement: Supplementary file 1 — Data S1: Supporting Information. [file JSP2-8-e70103-s001.docx]

STROBE Statement—checklist of items that should be included in reports of observational studies

|  | Item No. | Recommendation | Page  No. | Relevant text from manuscript |
| --- | --- | --- | --- | --- |
| **Title and abstract** | 1 | (*a*) Indicate the study’s design with a commonly used term in the title or the abstract | 1 | Quantitative Sensory Testing in an Observational Cohort of Adults with Chronic Low Back Pain |
|  |  | (*b*) Provide in the abstract an informative and balanced summary of what was done and what was found | 3 |  |
| Introduction | | | |  |
| Background/rationale | 2 | Explain the scientific background and rationale for the investigation being reported | 5 |  |
| Objectives | 3 | State specific objectives, including any prespecified hypotheses | 6 | The purpose of this manuscript is to present descriptive results of a series of QST measures collected as part of a large cLBP cohort study. |
| Methods | | | |  |
| Study design | 4 | Present key elements of study design early in the paper | 7 |  |
| Setting | 5 | Describe the setting, locations, and relevant dates, including periods of recruitment, exposure, follow-up, and data collection | 7 | Participants were enrolled by referral from clinicians, research registries, and community announcements between June 2020 and March 2024. The in-person enrollment visit took place at the University of Pittsburgh Department of Physical Therapy - Clinical and Translational Research Center. Participants were followed remotely for 12 months and were compensated for their participation incrementally at all timepoints. |
| Participants | 6 | (*a*) *Cohort study*—Give the eligibility criteria, and the sources and methods of selection of participants. Describe methods of follow-up  *Case-control study*—Give the eligibility criteria, and the sources and methods of case ascertainment and control selection. Give the rationale for the choice of cases and controls  *Cross-sectional study*—Give the eligibility criteria, and the sources and methods of selection of participants | 7 | Eligible participants were English-speaking adults with cLBP, defined as "back pain (in the space between the lower posterior margin of the rib cage and the horizontal gluteal fold) that persisted at least three months and resulted in pain on at least half the days in the past six months.”^20^ Participants were excluded if there they were 1) not identified in the University of Pittsburgh Medical Center (UPMC) Electronic Health Record (EHR) System, 2) participating in a masked intervention study for LBP, and/or 3) had a medical condition that would place the participant at increased risk or preclude them from complying with study procedures.^21; 22^ |
|  |  | (*b*) *Cohort study*—For matched studies, give matching criteria and number of exposed and unexposed  *Case-control study*—For matched studies, give matching criteria and the number of controls per case |  |  |
| Variables | 7 | Clearly define all outcomes, exposures, predictors, potential confounders, and effect modifiers. Give diagnostic criteria, if applicable | 8-9 |  |
| Data sources/ measurement | 8* | For each variable of interest, give sources of data and details of methods of assessment (measurement). Describe comparability of assessment methods if there is more than one group | 8-9 |  |
| Bias | 9 | Describe any efforts to address potential sources of bias |  |  |
| Study size | 10 | Explain how the study size was arrived at | 7 | Participants were told that the four QST procedures would cause some mild discomfort/pain, but that none of the procedures were harmful in any way. Some participants, such as those reporting cardiovascular disease, Raynaud’s syndrome, or diabetic neuropathy, were excluded from tests that involved immersion of the hand in cold water. Participants were allowed to opt out of any or all procedures. The number of participants that completed the QST tasks was 645 for CPM, 958 for PPT, 971 for TS, and 653 for the cold-water tolerance task. |

Continued on next page

| Quantitative variables | 11 | Explain how quantitative variables were handled in the analyses. If applicable, describe which groupings were chosen and why | 9 |  |
| --- | --- | --- | --- | --- |
| Statistical methods | 12 | (*a*) Describe all statistical methods, including those used to control for confounding | 9 | Measures of central tendency were computed: means and standard deviations (SDs) for continuous data, and percentages or counts for dichotomous data such as lingering pain/aftersensations. The data for the overall group are also presented as histograms to illustrate variability in QST results. |
|  |  | (*b*) Describe any methods used to examine subgroups and interactions | 10 | The results are presented for the overall group and stratified by sex at birth (male and female) and by age (<60 and ≥60 years) in **Table 1**. For various reasons described earlier, not all 1,007 participants received all four QST procedures. |
|  |  | (*c*) Explain how missing data were addressed |  |  |
|  |  | (*d*) *Cohort study*—If applicable, explain how loss to follow-up was addressed  *Case-control study*—If applicable, explain how matching of cases and controls was addressed  *Cross-sectional study*—If applicable, describe analytical methods taking account of sampling strategy |  |  |
|  |  | (*e*) Describe any sensitivity analyses |  |  |
| Results | | | | |
| Participants | 13* | (a) Report numbers of individuals at each stage of study—eg numbers potentially eligible, examined for eligibility, confirmed eligible, included in the study, completing follow-up, and analysed | 10 | Table 1  The results are presented for the overall group and stratified by sex at birth (male and female) and by age (<60 and ≥60 years) in **Table 1**. For various reasons described earlier, not all 1,007 participants received all four QST procedures. |
|  |  | (b) Give reasons for non-participation at each stage |  |  |
|  |  | (c) Consider use of a flow diagram |  |  |
| Descriptive data | 14* | (a) Give characteristics of study participants (eg demographic, clinical, social) and information on exposures and potential confounders | 10-13 | Tables 1 |
|  |  | (b) Indicate number of participants with missing data for each variable of interest |  |  |
|  |  | (c) *Cohort study*—Summarise follow-up time (eg, average and total amount) |  |  |
| Outcome data | 15* | *Cohort study*—Report numbers of outcome events or summary measures over time |  |  |
|  |  | *Case-control study—*Report numbers in each exposure category, or summary measures of exposure |  |  |
|  |  | *Cross-sectional study—*Report numbers of outcome events or summary measures |  |  |
| Main results | 16 | (*a*) Give unadjusted estimates and, if applicable, confounder-adjusted estimates and their precision (eg, 95% confidence interval). Make clear which confounders were adjusted for and why they were included | 10-13 | Table 1, Figures 1-4 |
|  |  | (*b*) Report category boundaries when continuous variables were categorized |  |  |
|  |  | (*c*) If relevant, consider translating estimates of relative risk into absolute risk for a meaningful time period |  |  |

Continued on next page

| Other analyses | 17 | Report other analyses done—eg analyses of subgroups and interactions, and sensitivity analyses |  |  |
| --- | --- | --- | --- | --- |
| Discussion | | | | |
| Key results | 18 | Summarise key results with reference to study objectives | 13-14 |  |
| Limitations | 19 | Discuss limitations of the study, taking into account sources of potential bias or imprecision. Discuss both direction and magnitude of any potential bias | 14-15 |  |
| Interpretation | 20 | Give a cautious overall interpretation of results considering objectives, limitations, multiplicity of analyses, results from similar studies, and other relevant evidence | 15 |  |
| Generalisability | 21 | Discuss the generalisability (external validity) of the study results |  |  |
| Other information | |  | | |
| Funding | 22 | Give the source of funding and the role of the funders for the present study and, if applicable, for the original study on which the present article is based | 18 | The Back Pain Consortium Research Program is administered by the National Institute of Arthritis and Musculoskeletal and Skin Diseases. This research was supported by the National Institutes of Health through the NIH HEAL Initiative under award number U19AR076725-01. The content is solely the responsibility of the authors and does not necessarily represent the official views of the National Institutes of Health or its NIH HEAL Initiative. |

*Give information separately for cases and controls in case-control studies and, if applicable, for exposed and unexposed groups in cohort and cross-sectional studies.

**Note:** An Explanation and Elaboration article discusses each checklist item and gives methodological background and published examples of transparent reporting. The STROBE checklist is best used in conjunction with this article (freely available on the Web sites of PLoS Medicine at http://www.plosmedicine.org/, Annals of Internal Medicine at http://www.annals.org/, and Epidemiology at http://www.epidem.com/). Information on the STROBE Initiative is available at www.strobe-statement.org.
